# The Molecular Fingerprint of High Grade Serous Ovarian Cancer Reflects Its Fallopian Tube Origin

**DOI:** 10.3390/ijms14046571

**Published:** 2013-03-25

**Authors:** Mirjana Kessler, Christina Fotopoulou, Thomas Meyer

**Affiliations:** 1 Max Planck Institute for Infection Biology, Department of Molecular Biology, Charitéplatz 1, 11017 Berlin, Germany; E-Mail: Kessler@mpiib-berlin.mpg.de; 2 Department of Gynecology, Charité, Campus Virchow Clinic, University Hospital Augustenburger Platz 1, 13353 Berlin, Germany; E-Mail: christina.fotopoulou@charite.de

**Keywords:** serous ovarian cancer, fallopian tube, p53 signature, STIC, cellular transformation, cancer stem cells (CSC), tumor microenvironment

## Abstract

High grade serous ovarian cancer (HGSC), the most lethal and frequent type of epithelial ovarian cancer (EOC), has poor long term prognosis due to a combination of factors: late detection, great metastatic potential and the capacity to develop resistance to available therapeutic drugs. Furthermore, there has been considerable controversy concerning the etiology of this malignancy. New studies, both clinical and molecular, strongly suggest that HGSC originates not from the surface of the ovary, but from the epithelial layer of the neighboring fallopian tube fimbriae. In this paper we summarize data supporting the central role of fallopian tube epithelium in the development of HGSC. Specifically, we address cellular pathways and regulatory mechanisms which are modulated in the process of transformation, but also genetic changes which accumulate during disease progression. Similarities between fallopian tube mucosa and the malignant tissue of HGSC warrant a closer analysis of homeostatic mechanisms in healthy epithelium in order to elucidate key steps in disease development. Finally, we highlight the importance of the cancer stem cell (CSC) identification and understanding of its niche regulation for improvement of therapeutic strategies.

## 1. Introduction

Epithelial ovarian cancer (EOC) represents a very heterogeneous disease with widely varying histology, pathogenesis and clinical presentation, resulting in greatly different outcomes. It is the fourth leading cause of cancer deaths among women in industrialized countries. According to the classification of the International Federation of Gynecology and Obstetrics (FDA, USA) in more than 75% of patients, initial diagnosis is made at stage III or IV of the disease. Consequently, despite advances in surgery and therapy, overall prognosis remains relatively poor, with a 10 year relative survival rate of less than 35% [1,2]. Chemotherapy resistance mechanisms, pre-existing resistant clones at initial presentation that survive suboptimal surgery, and unknown, molecular fingerprints inducing and maintaining invasion and metastasis are some of the underlying mechanisms responsible for this unfavorable prognosis.

**Figure 1:**
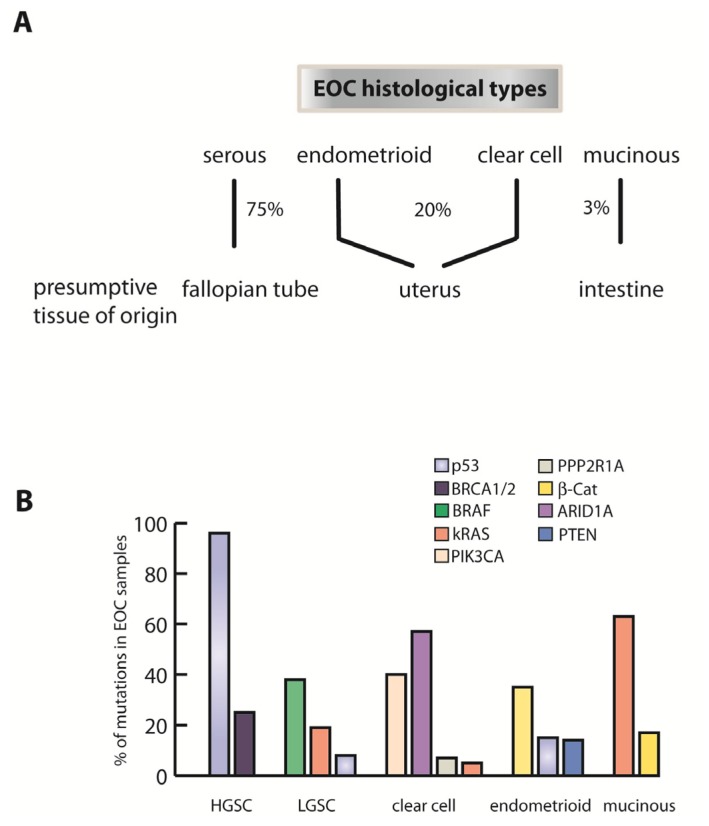
Epithelial ovarian cancer is heterogeneous in origin and molecular characteristics. (**A**) Serous ovarian cancer, the most prevalent type of epithelial ovarian cancer (EOC), originates in the fallopian tube, while other histological types are presumed to originate from the uterus and gastrointestinal tract; (**B**) The diversity of frequent somatic mutations in different types of EOC reflects the great divergence in histopathology and clinical presentation [5,8,11].

Recent pathological and genomic findings strongly suggest that many ovarian cancers are derived from non-ovarian tissues. The different histotypes share few molecular similarities and present with such different pathology that pelvic or peritoneal cancer may be a more suitable definition of the disease. For example, the distal fallopian tube has been identified as a source of high-grade serous ovarian cancers, the deadliest and most prevalent form [3]. The other histological subtypes of ovarian cancer consist of clear cell and endometrioid cancer, as well as mucinous ovarian cancer, which are proposed to have origins in the uterine and gastrointestinal tract, respectively (Figure 1A). In comparison to serous ovarian cancer, other histological subtypes present a distinct molecular and genetic profile (Figure 1B). This is in congruence with clear differences in morphology. For example, clear cell carcinoma was found to have a high incidence of PI3 kinase mutations and ARID1A [4,5], endometrioid tumors are characterized by high proportions of β-catenin mutations [6] and mucinous cancers predominantly show mutations in the Ras pathway [7]. However, for all subtypes, the precise mechanisms of the initial molecular transformation and spread of malignant cells to the ovary remain obscure.

Serous ovarian cancers vary in differentiation status, cytological features and genetic fingerprint, and are classified as Type I and Type II cancers: Low Grade Serous Carcinoma (LGSC) (Type I) and High Grade Serous Carcinoma (HGSC) (Type II). LGSCs exhibit a gradual, slow transition from benign to malignant state, involving multiple somatic mutations in K-Ras, BRAF, and PPP2R1A [8], thereby altering multiple cell growth and cell division related signaling pathways, which lead to a stepwise transformation and development of malignancy. HGSC on the other hand, are genetically diverse, fast-growing and -disseminating aggressive tumors, which bear only a small number of core somatic mutations resulting in a *de novo*, diffusely disseminated peritoneal disease. Tumor suppressor p53 inactivation is detected in more than 95% of cases and there is frequent inactivation of the BRCA1/2 DNA damage repair pathway within an otherwise greatly heterogeneous genetic background of sporadic HGSC patients. Around 10% of total HGSC cases are diagnosed in women with hereditary BRCA1/BRCA2 germ line mutations [9], while the remaining 90% are sporadic.

Despite increasing efforts in terms of more radical surgery and more efficient systemic treatment, survival of women with epithelial ovarian cancer has changed little since platinum-based treatment was introduced over 30 years ago. Therefore, understanding the biological origins and early stages of the disease remains imperative. Greater efforts should be made in the area of disease prevention, especially in BRCA mutation carriers. In this respect, it is necessary to elucidate the role of the fallopian tube as the putative tissue of origin of ovarian cancer. Increasing evidence suggests that the role of the ovary in the carcinogenesis process is limited to creating an unfavorable microenvironment, by releasing toxic substances into the follicular fluid after ovulation, that create local inflammatory processes which affect the epithelium of fallopian tube fimbriae, leading to neoplastic changes and finally malignant transformation [10]. Thus, understanding the regulatory mechanisms and signaling pathways in fallopian tube epithelium is becoming a focal point of serous ovarian cancer research. In this paper we provide an overview of the current findings about the genetic makeup and cellular phenotype of ovarian cancer tissue, and focus on the importance of the fallopian tube in its etiology.

## 2. Fallopian Tube as Extra-Ovarian Tissue of Origin of Serous Ovarian Cancer

**Figure 2:**
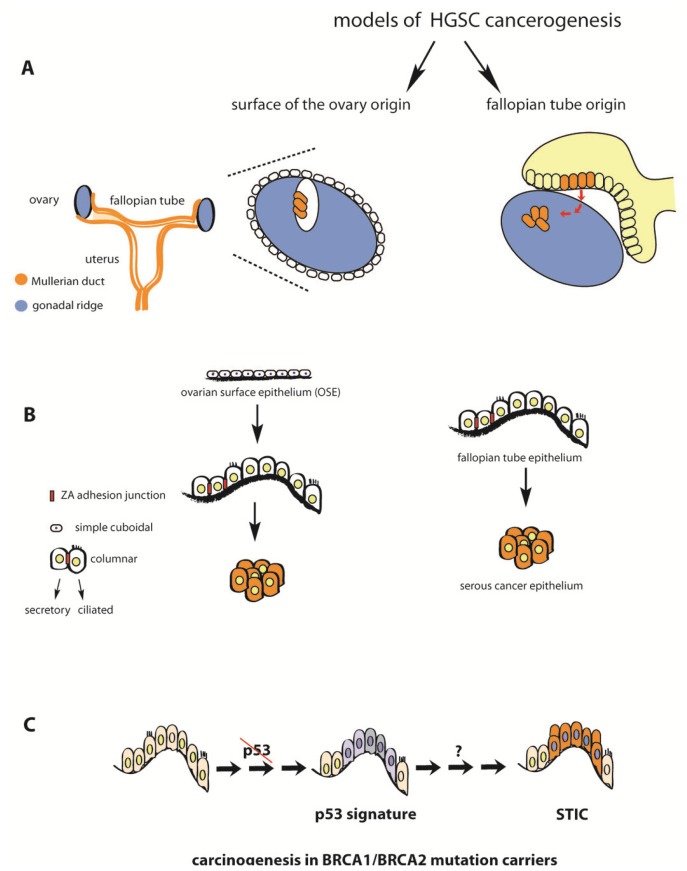
High grade serous ovarian cancer (HGSC) originates from fallopian tube mucosa. (**A**) The two models of ovarian cancer origin, comparing the old metaplasia model of OSE and the model with fallopian tube fimbriae as the source of malignant cells. Different colors, (blue = ovary; and orange = fallopian tube) depict the different embryonic origin of the tissues as well as differences in cellular phenotype. Notably, malignant cells of HGSC also have a Müllerian phenotype (orange); (**B**) An ovarian origin of serous ovarian cancer would require a 3-step process (middle row), involving first the conversion of simple cuboidal into more complex columnar epithelium, followed by de-differentiation during tumor development, whereas the fallopian tube model proposes a direct, one-step malignant transformation of fallopian tube secretory cells; (**C**) Stepwise development of STICS in the fallopian tube epithelium involves p53 inactivation, followed by additional genomic changes (e.g., BRCA mutations).

No ovarian precursor lesions of HGSC have been identified so far, which should theoretically be the case if ovarian cancer originated exclusively from the ovarian surface. Instead, multiple lines of evidence point to the fallopian tube as the tissue of origin. During embryonic development, the primordial structure known as the Müllerian duct gives rise to the proximal two thirds of the vagina, the cervix, uterus and fallopian tubes, whereas the ovaries originate from the mesoderm which also forms the urinary tract (Figure 2A). This is reflected in profound differences in the cellular phenotype and characteristics of the finally developed epithelial tissues of the adult. In this context, the fact that whole genome expression profiling of HGSC samples showed a significant correlation with the expression patterns of normal fallopian tube epithelium provides important clues about its putative origin [12].

Further molecular evidence was provided by screening of serous ovarian cancer cell lines, which examined the significance of individual genes for the survival and proliferation of cancerous cells by RNAi-loss-of-function analysis. A genome-wide screen of pooled shRNAs in 25 ovarian cancer cell lines identified the transcription factor PAX8 (paired box gene 8), which is amplified in primary high grade ovarian tumors, as essential for survival and proliferation [13]. Pax8 belongs to the family of homeobox transcription factors, proteins which are uniquely positioned upstream in the cascade of molecular events which determine commitment of cells towards a particular developmental route. It is essential for the development of the lower genital tract and also plays a role in the early cell fate determination of the thyroid gland and kidney [14]. In the adult, the secretory cells of the fallopian tube epithelial monolayer express high levels of PAX8, but neighboring ciliated cells do not, and neither do any cells on the surface of healthy ovaries [15].

Data from other malignancies indicate the existence of a common mechanism of carcinogenesis whereby transcription programs controlled by “master regulator” genes that are transiently active during early development, are incorporated into the survival repertoire of malignant cells (e.g., MITF for melanoma and SOX2 for colon). Therefore it is tempting to postulate that the requirement for PAX8 for the survival and proliferation of ovarian cancer cell lines represents exactly such a regulatory mechanism. The absence of PAX8 in the adult ovary also strengthens the argument that HGSC originates in the fallopian tube mucosa, from where it spreads to the ovary. Otherwise, epithelial ovarian surface cells would have to undergo metaplastic transformation, and start expressing PAX8 and other downstream targets prior to malignant transformation.

A tubal origin of ovarian carcinoma is also supported by several histological and pathological observations. The normal ovarian surface epithelium (OSE) is a simple monolayer of squamous to cuboidal epithelium which expresses no specific markers of tissue differentiation (Figure 2B). It has only rudimentary tight junctions and cell-to-cell adhesion is regulated mainly by desmosomes, thus it is attached to the underlying basal lamina only loosely. Compared to other mucosal epithelia it expresses very low levels of E cadherin, and there is no expression of CA125 which is abundant in the surrounding extra-ovarian mesothelium, the epithelial layer which covers the abdominal cavity and reproductive organs [16,17]. In comparison, the fallopian tube epithelium is a monolayer of fully differentiated columnar, secretory and ciliated epithelial cells, which are interconnected by complete adhesion junction belts, the zonula adherens, and express high levels of E cadherin. Thus, a model by which ovarian cancer arises from OSE assumes the existence of transformation mechanisms which trigger the transition from simple cuboidal epithelium into more complex, tightly organized and more differentiated epithelium with many features of fallopian tube mucosa, which is contradictory to the accepted paradigm of malignant transformation.

Low grade serous cancer is frequently confined to the ovary without spread in the peritoneal cavity and more often diagnosed in earlier stages, and thus, until recently, widely accepted to develop from ovarian cortical cysts which undergo Müllerian metaplasia. In the process of stepwise transformation, these genetically stable cancers presumably arise from cysts converted to benign cyst adenomas and then to borderline tumors. Nevertheless, a detailed histological study of 178 patient samples of adnexal serous tumors, cyst adenomas, borderline tumors, and control samples of non-neoplastic ovarian tissue provided compelling evidence that ovarian cortical cysts also originate from fallopian tube fimbrial cells that have formed inclusion cysts, as no histological proof could be found in support of the Müllerian metaplasia hypothesis [18]. Two types of inclusion cysts were detected in the ovary: 22% were of ovarian epithelial origin, confirmed by calretinin staining, while 78% contained PAX8-positive secretory cells as well as ciliated cells, confirmed by tubulin staining, similar to normal tubal epithelium. Importantly, no cysts were found to contain a mixture of cell types which would be expected if metaplasia was a result of random conversion of ovarian surface epithelial cells. Instead, the presence of ciliated cells typical of fallopian epithelium in cortical cysts followed by gradual reduction in their frequency during the steps of malignant transformation speaks in favor of a model whereby the physiological tubal mucosa migrates to the ovary cortex, perhaps during the wound healing process following follicle rupture, and becomes encapsulated, forming an inclusion cyst. During malignant transformation, gradual expansion of secretory cells then occurs at the expense of ciliated cells in a step wise reduction of tubulin-positive cells from the epithelial inculsion cyst (30%) via serous cystadenoma (10%) and borderline tumor (5%) to complete absence in low grade serous cancer. As no cilia are found in malignant tissues of either histological types of serous ovarian cancer (LGSC and HGSC), it is difficult to reconcile how conversion of OSE cells into fallopian tube cells would first lead to the creation of both ciliated and secretory cells only for the former ones to be lost at later stages of the process (Figure 2B).

## 3. Serous Tubal Intraepithelial Carcinoma (STIC) in Asymptomatic BRCA Mutation Carriers and Sporadic HGSC Cases

BRCA1 and BRCA2 germline mutations are responsible for the vast majority of cases of hereditary ovarian cancer, which represents approximately 10% of all cases. The lifetime risk of developing ovarian cancer is estimated to be 54% and 23% for carriers of BRCA1 and BRCA2 mutations, respectively (King *et al*., 2003). However, BRCA inactivation is also frequently identified in sporadic cases of HGSC, as reported by the Cancer Genome Atlas Research Network [19], resulting in the term “BRCAness”, to describe the genetic profile of sporadic cases that resemble the profile of cancers from germline mutation carriers. In addition to BRCA1 and BRCA2 germline mutations, the BRCA1 promoter was found to be hypermethylated and therefore epigenetically silenced in 56 of 489 sporadic cases (11.5%). Despite the severe hereditary burden for BRCA1/BRCA2 mutation carriers to develop ovarian cancer, histological examination of tissue from prophylactic salpingo-oophorectomy failed to find any notable differences in the incidence of premalignant lesions of the ovarian epithelium compared to controls. On the contrary, the procedure revealed the existence of *in situ* malignant tumors (serous tubal intraepithelial carcinomas, STICs) in the fallopian tube mucosa in up to 10% of cases. This finding, independently confirmed in numerous prospective studies from different clinical centers [20–25], presents striking *in vivo* evidence that early “ovarian cancer” resides in the fallopian tube prior to migration to the neighboring ovary or peritoneum. STICs have recently been confirmed as putative precursors of HGSCs based on findings demonstrating identical TP53 mutations indicative of a clonal relationship between the two malignancies [26]. Also, STICs express several potential oncogenes frequently found in HGSC, further linking both lesions [18].

In sporadic cases of HGSC, analysis of malignant samples after surgery for advanced stage ovarian cancer revealed the existence of STICs within the fallopian tube in 67% of cases, 92% of which were confined to the fimbriated end [27]. These findings were confirmed by Kindelberger [28]. Other studies revealed a significant involvement of the fallopian tube in primary peritoneal serous carcinoma as well, with 47% of completely inspected tubes containing STICs [29]. The presence of STICs in fallopian tubes of patients with ovarian and peritoneal cancers could possibly explain the causative factor of primary cancers of the peritoneum, in the same way as fimbrial epithelium gives rise to high grade serous cancers of the ovary.

However, the discovery of STICs, although important, does not resolve the question of HGSC etiology, as it already represents an early malignant stage of the disease. Furthermore, some important distinctions between STIC and HSGC remain to be addressed in order to establish a causal relationship. Since STICs are non-invasive carcinomas, further genetic changes and/or alterations in cellular phenotype must occur prior to the transition to clinically recognizable HGSC. Due to the lack of substantial histological data sets of fallopian tube mucosa from healthy women without a hereditary predisposition to HGSC, it remains unclear if STICs always precede HGSC. Also, although pathology studies confirmed extensive tubal involvement in sporadic and hereditary HGSC cases, the clinical picture is not uniform, as there is still a proportion of ovarian and peritoneal cancers without detectable STICs. Thus, it is possible that the fallopian tube is not the tissue of origin in all cases of HGSC, or that mechanisms other than STICs can lead to malignant transformation. For example, some HGSCs could develop from ovarian cortical cysts [30], although they still might originate from the epithelium of the fimbrium, similar to the malignant transformation model of LGSC proposed by Li and colleagues, as described above [18].

The low accessibility of the fallopian tube by conventional non-invasive methods represents a serious limitation to the success of early screening and detection programs for HGSC. Fallopian tubes are difficult to visualize via transvaginal ultrasonography (TVUS), and patency tests, either by hysterosalpingography or laparoscopy, provide information only about the passage of liquid through, but not about putative cellular pathology within the lumen. Thus, new developments in medical imaging are necessary to enable programs for early serous cancer detection and prevention. However, resolving the role of the fallopian tube in the etiology of ovarian cancer will require prospective clinical studies designed to detect malignancy at early stages. A study in Canada focused on non-invasive screening of symptomatic women, with a combination of serum determination of the tumor marker CA-125 and TVUS [31]. In a group of 1,455 women, a total of 22 gynecological cancers were detected, 9 of which were classified as HGSC. Interestingly, 7 of these, which were localized outside the ovaries, had a relatively low CA-125 burden and no or very minor abnormalities detectable by TVUS on the ovarian surface.

On the other hand, a number of cell biological questions remains to be resolved in order to elucidate the cascade of events in the fallopian tube epithelium which lead to STIC formation. Further histological analysis is needed in order to identify precancerous lesions and molecular changes in the epithelium that favour malignant transformation. So far, immunohistochemistry for molecular markers revealed dysplastic morphological changes in the fallopian epithelium of BRCA1/BRCA2 mutation carriers compared to healthy controls, characterized by p53 positive nuclei foci, increased numbers of proliferative Ki67 expressing cells and also reduced numbers of p21 and p27 positive cells [32]. The observed antibody staining signature was always restricted to secretory cells and did not include ciliated cells and is more frequent in patients who develop STICs than in controls. Thus, p53 “signatures” in fallopian tube epithelium (Figure 2C) are postulated to be a form of cellular atypia *in vivo*, a benign alteration of secretory cells that provide the basis for malignant transformation and development of ovarian cancer.

## 4. P53 Inactivation Is a Necessary but not Sufficient Step in the Etiology of HGSC

Given the prevalence of p53 mutations in HGSC [33], it is clear that signaling pathways regulated by this tumor suppressor are of essential importance for the initial stages of cellular alterations which eventually lead to transformation. However, there are doubts if p53 signatures have real prognostic value when assessing the risk for future cancer development.

It is known that, p53 activation, marked by its accumulation in the nucleus, belongs to the central response to genotoxic stress in healthy cells [34] and p53 signatures are also found in the epithelium of healthy women. BRCA-mutated but still healthy women, as well as non-mutated non-neoplastic controls, have been shown to have the same frequency of p53 foci in the fimbrial epithelium [35]. Still, there was an increase in the number of p53 signatures in tubes containing STICs. Also, mutational analysis of DNA retrieved by laser-targeted microdissection of p53 foci showed that the majority of them (57%) contained mutated p53 alleles, while all samples from tubes containing STICs were p53 mutated. It appears that the fimbrial epithelium is susceptible to p53 accumulation but that additional genotoxic events and mutations are necessary for malignancy to occur. Interestingly, women with Li Fraumeni syndrome do not have increased rates of ovarian cancer. This rare genetic condition, inherited in a dominant autosomal fashion, where one copy of the p53 gene is inactivated in the germline, dramatically increases the risk for developing a number of malignancies, such as breast cancer, brain cancer, leukemia, soft tissue sarcomas *etc*. Risk is mediated by the relatively high probability of a “second hit” and inactivation of the remaining p53 allele due to environmental factors, which is sufficient to trigger malignant transformation. Still, loss of p53 function in the fallopian tube epithelium alone is not sufficient to cause transformation. Although the examined epithelium of Li Fraumeni syndrome mutation carriers contained increased numbers of p53 signatures compared to healthy controls, on the same level as HGSC patients’ samples [36], no associated malignancy was detected. Thus, it can be concluded that p53 inactivation is a necessary but clearly not sufficient step in transformation and additional genetic alternations are needed to trigger development of the disease. In the case of BRCA1 germline mutation carriers, this is achieved by additional genomic instability, caused through defects in the DNA repair machinery together with the loss of p53 function. Consequently, there is a dramatic increase in the risk of developing serous ovarian cancer. Therefore, the prevalence of p53 mutations in the tissue samples of HGSC patients do not prove its causality in disease etiology, but may rather be a conserved molecular mechanism which is modified in cancer cells at some stage of the transformation process, and preserved, due to the competitive advantage it confers upon HGSC cells. Since the vast majority of HGSCs are diagnosed at an advanced stage of the disease, genotyping of the tumor does not provide any information about when a particular mutation occurred. The fact that the fallopian mucosa frequently harbors aberrant p53 phenotypes provides an attractive model of the environment prone to conversion to STICs and finally HGSCs, but further studies are required before a model of p53 signatures as first precancerous lesion can be accepted as definite precursor of serous ovarian cancer.

## 5. Modulation of DNA Repair in Carcinogenesis and Mechanisms of Chemoresistance

Double stranded DNA break repair mechanisms by homologous recombination (HR) represent one of the most significant checkpoints for maintaining genome stability. Failure to efficiently correct simultaneous disruption of both strands promotes chromosomal rearrangements and thus further instability, driving the cell towards uncontrolled growth and transformation [37].

**Figure 3:**
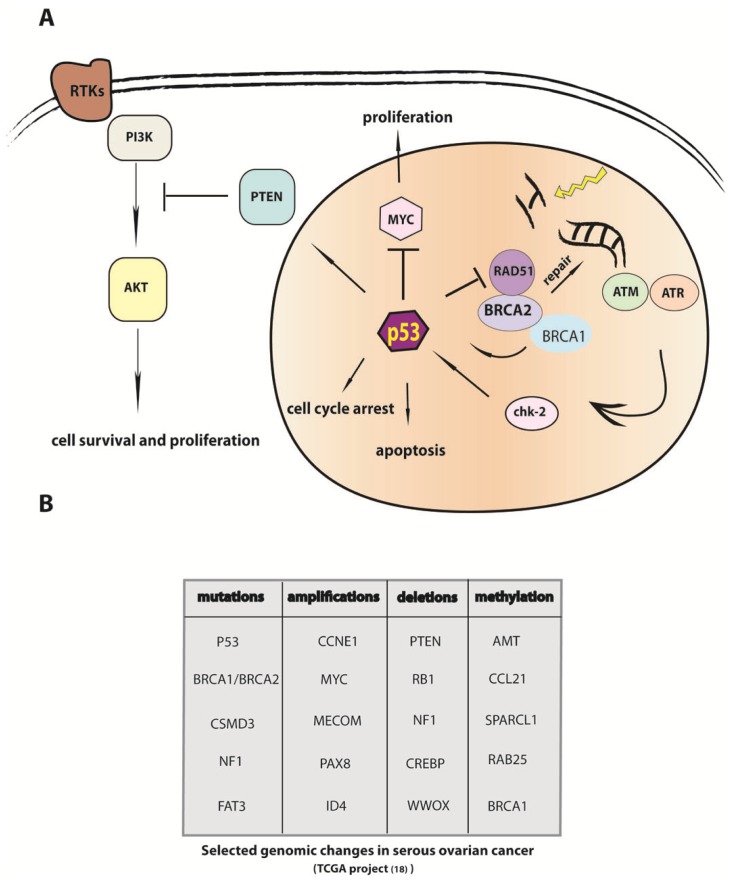
P53 and BRCA regulation of DNA repair represents a central component of altered cellular function in serous ovarian cancer. (**A**) BRCA proteins are activated in response to genotoxic stress via the ATM/ATR pathway and required for homologous recombination, while p53 protects cells from defective BRCA1 function by triggering cell cycle arrest and apoptosis. A combination of p53 and BRCA mutations, seen frequently in BRCA mutation carriers, thus allows several mechanisms to drive cell fate towards malignant transformation, e.g., myc overexpression or akt kinase signaling; (**B**) The most prominent genomic changes found in serous ovarian cancer patients ( The Cancer Genome Atlas TCGA study [19]). Their function was altered by different genetic molecular mechanism: somatic mutations, changes in copy number and expression level by methylation.

However, although deficiencies in homologous recombination mechanisms play a central role in the initiation of cellular transformation in HGSC, the progression of the disease at later stages, especially the development of resistance to therapy, appears to be dependent on the partial recovery of HR function in cancer cells. Elevated HR activity has been shown to promote aneuploidy and chromosome rearrangements [38]. Therefore, cancer cells with their robust capacity to overcome DNA breaks have a competitive advantage, survive and further diversify in response to therapeutics which mainly work as DNA damage inducing agents. In healthy cells, the tumor suppressors p53 and BRCA1/BRCA2 have a complementary role in maintaining DNA helix integrity (Figure 3A). Upon exposure to genotoxic stress, p53 inhibits homologous recombination by binding directly to RAD51 protein and also suppressing the expression of the *rad51* gene [39]. Also in BCRA1/BRCA2 mutant mouse embryos, which are deficient in homologous repair, there is no increase in malignancies, as might be expected, but instead a reduced rate of cell proliferation due to the compensatory activation of p53 [40], and a subsequent increase in cell cycle arrest through downstream expression of p21 [41].

This may explain why patients with the BRCA1/BRCA2-mutant form of HGSC have slightly better long-term survival and a longer remission period compared to sporadic cases [42]. This difference is thought to arise mainly due to a better response to platinum-based chemotherapy and PARP inhibitors, although this needs to be confirmed by additional trials. Indeed the importance of functional HR in malignant tissue for long term cancer progression has been demonstrated in a group of 50 patients whose HR status was confirmed by RAD51 assay prior to chemotherapy [43]. All patients received chemotherapy and PARPi treatment and during the 14 month follow-up, patients deficient in HR had a better response and long term prognosis than patients from the HR competent group. In addition Norquist and colleagues [44] reported the phenomenon of secondary somatic mutations, restoring the protein function, in hereditary BRCA1/BRCA2 patients who exhibit chemoresistance to platinum and PARPi. Although the detailed molecular mechanisms of acquired chemoresistance are yet to be fully characterized, the dependency of late tumors on functional HR mechanisms is considered to be a promising aspect of ovarian tumor biology for the development of new therapeutics.

## 6. Fallopian Tube Epithelium in BRCA1/2 Mutation Carriers: Searching for the Molecular Fingerprint of Carcinogenesis

Given the high risk BRCA mutation carries to develop HGSC, as indicated by the appearance of STICs, elucidating how the fallopian tube epithelium of carriers differs from controls on a molecular and cellular level could give valuable insight into the original cause and mechanism of cellular transformation. Indeed, a study of the genome wide expression profile of tubal epithelium in BRCA1 carriers confirmed that there are significant differences in the expression patterns of analyzed epithelial cells compared to controls [45].

The fallopian epithelium mucosa is exposed to immense physiological changes, driven by cyclically altering hormone levels. This is reflected in major changes of gene expression patterns between follicular and luteal phases, irrespective of any BRCA mutation status. However, the epithelium of BRCA mutation carriers also has a different signature for genes involved in inflammation signaling and DNA damage response compared to the epithelium of unaffected women. The upregulation of CEBP-δ, NAMPT, GADD 45-β, and decrease in phospho-STAT3 found in mutation carriers is likely to cause altered cellular responses to environmental stimuli, increasing the long term probability of cellular transformation. Immunohistochemistry results to complement the microarray findings provided the first molecular markers by which BRCA1-affected mucosa can be distinguished from healthy tissue. Clearly further studies are needed to elucidate the extent of cellular changes in epithelium with a BRCA1 mutation and to identify the core mechanism which is responsible for the dramatic susceptibility of these cells to ovarian cancer development. Still, initial findings stress the importance of inflammatory signaling, stress response and cell cycle control. Since BRCA mutations have only limited penetrance, providing strong risk for future cellular transformation but no direct mechanism for imminent cancer development, it is clear that other etiological factors need to be taken into account in order to understand the molecular background of HGSC.

## 7. The Tumorigenic Effects of Oxidative Stress and Inflammation in the Fallopian Tube

In addition to the regular epithelial turnover in the genital tract, monthly ovulation is considered to be a major event triggering inflammatory signaling at regular intervals in both the ovary and the adjacent fallopian fimbriae. Evidence for a tumorigenic effect of ovulation as such comes from several different findings. A large prospective epidemiologic study, analyzing over 320,000 women in Europe over a decade, confirmed the protective influence of long-term usage of oral contraceptives with regard to life-long ovarian cancer risk [46]. Parity, and consequently a prolonged lack of ovulation for a year or more, is also known to reduce ovarian cancer risk by 29%, with each new pregnancy further reducing the rate by 8%. In contrast, late menopause, associated with ovulation for a longer time period is associated with a significantly higher risk for ovarian cancer.

The influence of ovulation as a cause of increased gonadotropin stimulation as well as oxidative stress and local inflammation due to follicle rupture was studied in an *in vivo* mouse model as well as an *in vitro* 3D baboon model of tubal epithelium. While ovulation-related spikes in FSH and LH concentration did not alter the proliferation rate of tubal epithelium, oxidative stress triggered an increase in phosphorylated γ-H2AX, a marker of double-stranded DNA breaks in the tubal epithelium. Postovulatory tubal epithelium in mice also contained increased numbers of infiltrating macrophages close to the site of ovulated oocytes [47]. Nevertheless, while ovulation is a monthly physiological event during the reproductive years of most women, the risk of developing ovarian cancer is only 1 in 72 (SEER program National cancer Institute USA). Clearly, additional genetic and environmental factors play a role in the etiology of sporadic ovarian cancer.

A breakthrough in our understanding of the molecular biology of ovarian cancer may depend on gaining a deeper insight into homeostasis, inflammatory signaling and stress responses of normal fallopian tube epithelium. Monthly inflammation is not the only source of inflammatory pressure on the tubal mucosa. Ascending bacterial infections often cause extensive inflammation and pathology. Although no direct causative relationship between infectious agents and ovarian cancer has been conclusively proven as yet, protracted inflammation must be considered as a putative factor contributing to carcinogenesis.

A large population study from Taiwan comparing 67,936 women with a history of pelvic inflammatory disease (PID) with 135,872 controls, found a more than two-fold increase in the risk for development of ovarian cancer later in life [48]. The risk was directly correlated with the number of PID episodes.

*Chlamydia trachomatis* (*Ctr*) is found in up to 25% of patients diagnosed with PID [49] and 57% of patients with salpingitis [50], causing inflammation, scarring and occlusion of the fallopian tube. Based on the extent and spectrum of molecular interactions that *Ctr*, an intracellular pathogen, establishes within the host cells, it could conceivably trigger long-term cellular changes in the epithelial layer of the tube, posing a putative separate risk factor for ovarian cancer. *Ctr* infection triggers extensive epithelial damage, marked by loss of cilia [51,52]. Increased expression of Toll-like receptor 2 (TLR2) [53], cytokine secretion and infiltration of T lymphocytes [54] are reported in infected tissue. Also, *Ctr ex vivo* infection causes increased epithelial proliferation and activates the Wnt signaling cascade, affecting paracrine regulation of homeostasis [55]. Complementary to this finding, numerous *in vitro* studies of *Ctr* and *Neisseria gonorrhoeae* confirm the interference of these pathogens with signaling pathways that are known to be altered in HGSC, e.g., PARP signaling [56], cell cycle control [57] and apoptosis [58]. However, a causative link between infection and carcinogenesis in the genital tract remains difficult to prove, with the limited number of epidemiological studies providing contradictory data.

Ness and colleagues [59] reported a significant difference in IgG antibody titers against *Ctr* elementary bodies and heat shock proteins (CHSP) in the serum of ovarian cancer patients compared to the control group, but failed to validate the finding in a larger, population-based group [60]. In a more recent patient cohort analyzed by Idahl *et al*. [61], the presence of IgG anti HSP60-1 antibodies in serum was positively associated with the incidence of HGSC. These conflicting findings might be partially explained by the often unreliable serology in response to *Chlamydia* infection. Since *Ctr* infection has a high prevalence in the general population and only a fraction of infections result in ascending inflammatory processes, there is a need to identify novel markers which could distinguish between uncomplicated lower tract infections and upper tract pathology. Recently *Ctr* antigens CT157, CT423, CT727 and CT396 were identified as markers of upper genital tract infection [62]. Thus, further epidemiological studies are necessary to validate these markers for detecting asymptomatic chronic salpingitis and explore a potential association between *Ctr* infection and the development of malignancy through gaining a better understanding of the pathogen–host interaction. Alternatively, pathogen-specific genetic and/or epigenetic fingerprints remaining after infections might help to trace possible etiological links with cancer initiation.

## 8. The Molecular Basis for Malignant Transformation in Fallopian Tube Epithelium: Analysis of Cellular Pathways

Realizing the importance of the fallopian tube in the process of carcinogenesis in HGSC, several *in vitro* studies have been designed to simulate the malignant transformation of the fallopian tube epithelial cells by targeting distinct pathways. Jazaeri and colleagues [63] focused on introducing p53, BRCA, HRas and Rb mutations, as well as overexpression of c-MYC and hTERT by retroviral integration. The two generated cell lines with the highest proliferation capacity *in vitro* were shown to generate tumors in the SCID mouse xenograft model *in vivo.* Histological analysis of malignant tissue revealed great similarities with ovarian cancerous tumors. Interestingly, attempts to reduce the number of components in the “oncogenic cocktail”, led to failed tumorigenesis *in vivo*, demonstrating that more profound alterations of the cellular regulatory networks are required to drive cancer formation.

Recently, Karst and colleagues succeeded in demonstrating the stepwise immortalization and subsequent malignant transformation of primary fallopian tube epithelial cells [64]. By introducing defined genetic elements and testing combinations of these, they demonstrated successful xenograft tumor models when HTERT and SV40 proto-oncogene expression was combined with either cMYC or oncogenic Ras. They also found complete *in vitro* transformation and *in vivo* tumor growth in the absence of the viral oncogene SV40 with a knock-down of p53 in the cells and CDK4 and shPP2A-B56γ mutation (the latter being well-known cellular targets of SV40 function). Comparative histological analysis of the generated tumors also showed a strong similarity to serous ovarian cancer tissue architecture, normally found in patients with extensive peritoneal involvement. Thus, primary fallopian tube epithelial cells appear to be a useful *in vitro* model for studying the development of HGSCs, using controlled genetic manipulations and defined experimental settings. Further expansion of similar studies to include a more detailed molecular analysis of molecular pathways based on gene candidates retrieved from HGSC patient samples, could be of crucial importance for illuminating key phases of the transformation process. In recent years, high-throughput technology generated data to make such studies comprehensive and feasible.

In addition to xenograft models for studying HGSC malignant transformation *in vivo*, there have also been attempts to demonstrate the fallopian tube origin of the malignancy in genetically tractable mouse models. In a recent study, Kim and colleagues found that combined knockdown of Dicer, an essential factor for production of mature miRNAs, together with PTEN, a negative regulator of PI3 kinase and therefore activator of Akt signaling, leads to formation of aggressive serous ovarian cancer, with 100% lethality between 6 and 13 months after birth [65]. Tumors originated exclusively from the fallopian tube, as demonstrated convincingly by histological analysis of early stages of the disease. These findings provide an important *in vivo* “proof of principle” for the fallopian tube model of ovarian carcinogenesis, regardless of considerable differences in morphology and physiology between mouse and humans. It also brought into focus additional cellular pathways, separate from core components of the DNA stress response and repair machinery, which could be essential for development of HGSC.

## 9. The Genomic Sequence of Ovarian Carcinoma and Implications for Understanding the Etiology of the Cancer Cell

The concept of the fallopian tube as tissue of origin of HGSC evolved as a hypothesis based on a combination of clinical findings in BRCA mutation carriers and histological and molecular similarities between healthy fallopian tube mucosa and malignant tissue. A detailed understanding of HGSC carcinogenesis and full acceptance of the fallopian tube paradigm, however, will depend on deeper insights into the cellular biology of both cancer tissue and fallopian tube. The individual molecular and cellular markers (e.g., PAX8 or p53 mutations) or morphological changes (cellular atypia, STICs) presented in detail above, have limited and descriptive value if taken out of context of cell-cell communication and regulation of growth and proliferation mechanisms within the tissue. In the last decade, research dealing with cancer genomics and cancer stem cells has emerged, which focuses on the analysis of signaling pathways, cellular mechanisms, and putative hierarchy in tumor samples, all of which should help to resolve the remaining questions about the origin of HGSC and offer new therapeutic strategies. Although many of the approaches dealing with cancer stem cells, epithelial mesenchymal transition and tumour dissemination are yet to find their way into clinical practice, they provide a valuable basis that should yield a therapeutic breakthrough in the future.

Despite the great heterogeneity in HGSC tissue samples, modern genomics has enabled large-scale analysis of the genetic changes behind the malignant phenotype, and discovery of common molecular patterns. In 2011, the Cancer Genome Atlas (TCGA) consortium completed a comprehensive genomic analysis of 489 serous ovarian carcinoma samples. This large scale project included DNA sequencing, gene copy number analysis, mRNA and miRNA profiling and determination of methylation changes. Parallel, complementary pipelines not only allowed the significance of known molecular markers to be tested, but also led to the identification of so far unknown players and altered cellular mechanisms. The results confirmed that inactivation of the p53 tumor repressor pathway represents a central component of nearly all analyzed tumors (96%) and a converging point of cellular changes which eventually lead to advanced tumor progression. Mutations of nine other genes occurred with lower but significant frequency, many of which were already linked to HGSC in previous studies, including BRCA1 and BRCA2, NF1, CDK12 and RB1 [66,67].

However, perhaps the most valuable new insight brought by TCGA analysis was an understanding of the complexity of regulation on a genomic level (Figure 3B). Only a minor fraction of alterations in cancer cells is due to classical mutations, e.g., changes in protein coding regions. Instead, the combination of structural genomic alterations, e.g., changes in copy number, and alterations in regulatory mechanism e.g., expression pattern and epigenetic modulation of gene activity, play a crucial role in cellular transformation. For example, the TCGA study confirmed a recurring pattern of a total of 8 gains and 22 losses within chromosomal bands many of which have been reported previously [68,69]. Downstream analysis of affected genes identified CMYC, CCNE1, MECOM, ID4, PAX8 and TERT as focal amplification peaks. Genomic regions encoding the well-known tumor suppressor genes PTEN, RB1 and NF1 were found to be frequently deleted in HGSC [70]. Integrated data from different experimental set ups (sequencing, methylation analysis, copy number and gene expression profiles) were pooled and subjected to network analysis revealing which biological pathways are altered in ovarian cancer tissue. DNA repair mechanisms as well as RB1, PI3K and NOTCH pathways were significantly modified in many ovarian cancer tissues. Moreover, the regulatory network of the transcription factor FOXM1, known for its role as an oncogene in basal cell carcinoma, glioblastoma, breast cancer and prostate cancer [71,72], was for the first time associated with ovarian cancer. In 87% of all HGSC cases, the FOXM1 signaling was altered, in congruence with the identified overexpression of the downstream target genes AurB, CCNB1, PLK1, CDC25 and BIRC5. In a more detailed follow-up study, *in vitro* models based on the ovarian cancer cell line OVCA433 showed a clear dependency of FOXM1 on upstream activation of MEK/ERK signaling, [73]. More data will be needed to elucidate the involvement of FOXM1 in serous ovarian cancer development and progression, but these findings are potentially important with regard to therapeutic strategies, since potent inhibitors of FOXM1 exist, which are known to have tumor suppressing capacity in other malignancies [74].

## 10. Regulation of Epithelial-Mesenchymal (EMT) Transition and Dissemination of HGSC

Aggressive spread of cancer tissue is another hallmark of HGSC which make it difficult to treat. Therefore, it is of pivotal interest to elucidate the cellular mechanisms that drive tumor growth as diseases progresses. A better understanding of fallopian tube epithelial homeostasis may be necessary to achieve this.

The majority of epithelial cancers undergo epithelial-mesenchymal transition (EMT) at some stage of disease progression. This is a core mechanism in tumorigenesis of epithelial tissues that leads to invasiveness and aggressive proliferation of cells. Loss of adhesion and polarity is mediated by suppression of E-cadherin expression and disassembly of adherens junctions. It has been shown that the ovarian cancer cell lines SKOV3 and OVCAR5 in response to stimulation by EGF exhibit a strong downregulation of E cadherin and increased invasion capability. Mechanism is dependent on induction of transcription factors Egr1 and Snail [75]. Many studies independently found a correlation between the EMT process and development of chemo resistant properties of the cancer. Direct contribution of transcriptional factors Snail and Slug in cisplatin resistance was found by Haslehurst and colleagues [76] in proteomic and transcriptional analysis of ovarian cancer cell lines and confirmed in a cohort study of clinical samples.

Also, induction of the EMT-related transcription factors snail and slug occurs downstream of other receptors, for example the endothelin A Receptor (EtAR), which is upregulated in primary tumors of patients resistant to platinum based therapy plus paclitaxel. Blockage of EtAR with zibotentan strongly reduced EMT and proliferation of EOC cell lines *in vitro* and tumor growth in a xenograft model *in vivo*, and resensitized cells to chemotherapy [77]. These are only a few examples where extracellular signaling pathways are shown to influence EMT transition in ovarian cancer. Ultimately, however, conversion of the cellular phenotype from epithelial to mesenchymal is an intracellular mechanism that is the end result of interfering developmental programs and environmental signals.

Maintenance of epithelial phenotype with strong Cdh1 expression is a property of differentiated and polarized cells. Events which change the intracellular environment towards a less differentiated status and pluripotency can also contribute to EMT. Thus, the stem cell marker nanog was found expressed in ovarian cancer cell lines and patients with advanced tumor stages, resistant to chemotherapy [78] Further, nanog was found to be an independent prognostic factor for disease survival of patients, increasing invasiveness and metastatic potential by direct suppression of Cdh1, caveolin-1, FOXO1, FOXO3a, FOXJ1 and FOXB1 [79].

Different scenarios could be envisaged which could initiate reprogramming of cells towards stemness during tumorigenesis. So far, it has been shown that nanog expression directly depends on p53 levels in the cell, which on its own is affected by expression of microRNA-214 [80]. This is only one example of the interconnectivity of cellular pathways, in this case linking DNA repair with cell fate determinants and EMT mechanisms. When applied to the situation within the premalignant epithelial layer, the loss of p53 frequently seen in tubal fimbriae should lead to ectopic expression of nanog in differentiated epithelial cells, potentially reversing their lineage commitment and life span. This and other similar observations from cancer cells are yet to be tested in non-malignant, pre-cancerous tubal epithelium in order to establish a clear causal relationship and determine their importance for the initial transformation process.

## 11. Notch Paracrine Signaling, Stemness, and Tumor Progression

Proliferation, regeneration and renewal in epithelium are regulated by complex mechanisms of cell-cell communication and paracrine signaling. During the last decade, in numerous different tissues (stomach, intestine, colon, skin, nervous system *etc*.), adult stem cells have been identified [81–83] which enable long term sustained renewal and generation of healthy, differentiated cells. They reside within a niche in close communication with the surrounding mucosa, and give rise to progeny which initiate the differentiation process, undergoing further divisions along the way. This cell fate determination process within the tissue is tightly regulated by a paracrine signaling network and integration of Wnt, Notch, BMP and Shh signaling cascades. Mechanisms of epithelial renewal of the fallopian tube remain obscure, as some evidence also points to the existence of pluripotency in the epithelial layer [84].

So far it is unclear if there is a relationship between healthy tissue stem cells and cancer stem cells which have been described in numerous malignancies. Cancer stem cells could originate from tissue-specific stem cells which have acquired further mutations and the capability to growth outside the niche independent of regulatory mechanisms. Alternatively, cancer stem cells could be the result of reprogramming and de-differentiation of normal somatic cells during transformation.

Recent data from both clinical studies and *in vitro* analysis of cell lines shows the importance of the NOTCH signaling pathway for the progression of HGSC and general survival of the patients. Increased expression levels of NOTCH3 correspond with significantly higher recurrence rates of cancer in affected patients and a shorter disease-free periods [85]. Overexpression of NOTCH3 leads to increased platinum resistance, whereas γ-secretase treatment, which inhibits intracellular transmission of the notch signal, restores sensitivity to therapy and depletes cancer stem cells in the tumor [86]. It remains to be seen if the effects of NOTCH3 inhibition can be attributed to the specific suppression of cancer stem cells within the tumor population. Also, more research is needed into the general mechanisms of notch paracrine signaling in the fallopian tube and the ovary, in order to establish its contribution to carcinogenesis in more detail.

## 12. Cancer Stem Cells in Proliferation and Survival of HGSC

**Figure 4:**
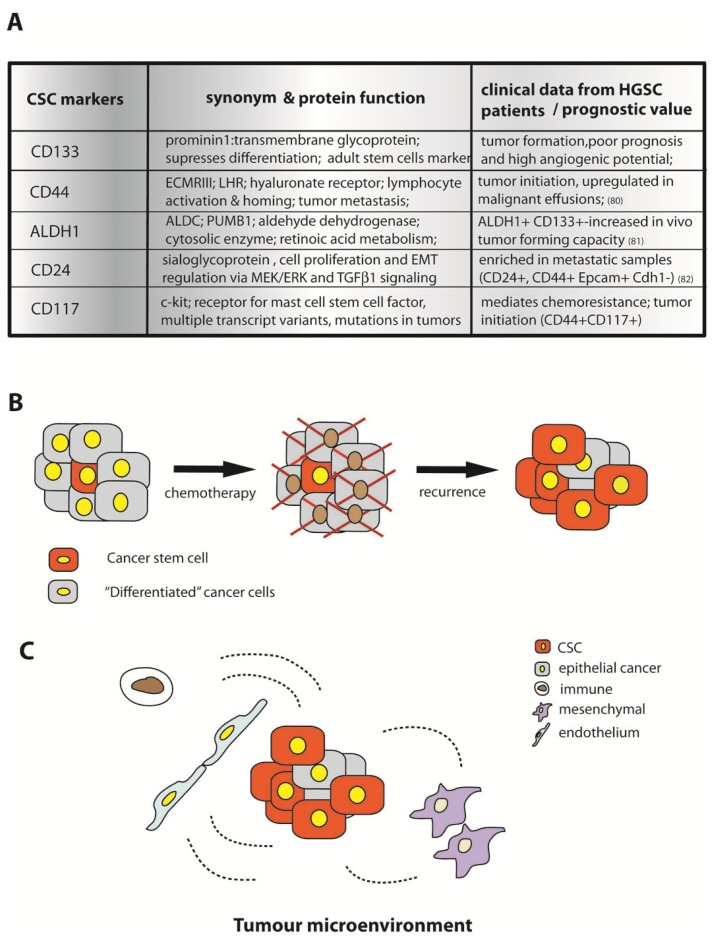
Ovarian cancer stem cells (CSC) are responsible for disease progression and acquisition of chemoresistance. (**A**) Candidate proteins identified as ovarian CSC markers; (**B**) Model of CSC-driven cancer progression: CSCs represent a small population of the initial primary tumor, which are resistant to chemotherapy. Following treatment, they give rise to more differentiated cells, which are then also resistant to therapeutics in the recurrent phase of the disease; (**C**) The tumor microenvironment, comprised of different cell types, influences the cancer tissue directly affecting the behavior of CSCs and tumor growth patterns.

Although EOCs appear to be of clonal origin, during the advancement of the tumor and its spreading not all cells have the same potential to initiate and sustain growth. It is evident that only a minor percentage of cells retrieved from malignant tissue *ex vivo*, shows clonogenic growth *in vitro* and can give rise to novel tumors in xenograft models *in vivo*. Cancer stem cells (CSCs) were first described in hematopoietic cancers, as a subpopulation of cells that is long-lived with self renewal capacity, differentiation potential and resistance to therapy. In ovarian cancer, CD44+CD117+ cells were identified as a subpopulation of cells from the primary tumor, with sustained capacity to initiate tumorigenesis in xenografts, in contrast to CD44-CD117- cells [87]. CD44+MyD+ positive cells isolated from patients also showed properties of CSCs *in vitro* and *in vivo*[88]. The number of CD44+ cells in the tumor corresponds with the stage of the disease, as the percentage of positive cells in histological samples rises from 6.3% in primary tumors to 18% in metastatic tissue in representative patients. The differential expression profile of CD44+ versus CD44- EOC cells revealed a strong upregulation of cytokeratin 18, β-catenin, and entire gene families involved in regulation of cell cycle and apoptosis. CSCs were found to exhibit functional, constitutively active NfkB signaling, probably mediated by the upstream TLR/MyD 88 pathway. As a consequence of these changes, cells develop resistance to the standard therapeutic drugs paclitaxel and carboplatin. Additional studies reported stemness potential in cells positive for CD133, aldehyde dehydrogenase isoform 1 (ALDH1), CD24+ and EpCAM+ [89,90] (Figure 4A).

The diversity of reported markers for ovarian cancer stem cells may be indicative of different origins of the cancer initiating cells. Alternatively, certain discrepancies in reported markers might be due to methodological differences in the design of the studies analyzing established ovarian cancer cell lines versus primary fresh material, selection criteria of patient samples *etc*. For example, it has recently been demonstrated that *in vitro* cultivation of fresh cells isolated from primary tumors in the presence of fetal calf serum leads to a gradual loss of the stem cell markers CD133, ALDH1, CD24, CD44 and CD117 [91]. Transplantation into the SCID mouse model led to recovery of CD133, and ALDH1 while other markers were permanently lost. However, a common factor in all of the studies dealing with presumptive cancer stem cells isolated from HGSC is their intrinsic capacity to drive tumor growth and disease progression via multiple mechanisms simultaneously. The importance of inflammatory signaling for the function of CSCs was demonstrated by Long *et al*. [92] who defined EOC stem cells as CD133+, expressing receptors for the chemokine signaling components CCL5 and receptors CCR1, CCR3 and CCR5. They showed that an autocrine chemokine signaling loop perpetuates the NFkB signaling cascade and increases the release of MMP9, which mediates invasiveness. This represents one potential mechanism by which cancer stem cells gain a competitive advantage over other cells from the tumor. For example expression of the endothelin-A receptor, mentioned previously for its role in chemoresistance and EMT induction, is essential for both ICAM1 upregulation, which ensures immune cell recruitment, and for proliferation of the chemoresistant CD133+ CSC population [93]. Thus CSCs appear to play an important role in all crucial steps of pathology development and disease spread, from modulation of the immune response, to angiogenesis, invasiveness and dissemination. In addition, their resistance to standard chemotherapeutical agents imposes great limitations on treatment options (Figure 4B). One of the great difficulties in translating emerging knowledge about ovarian cancer stem cells into efficient therapeutic strategies is our lack of fundamental understanding of their origin in healthy tissue and the regulatory mechanisms which control their niche in the tumor. For example, accounted differences in isolated CSC populations could be explained by different stages of stemness and differentiation. Further, numerous studies have reported a contribution of the individual components of the tumor microenvironment to the proliferation and survival of CSCs in selected patients. The malignant potential of the tumor is driven by epithelial cells, but mesenchymal and endothelial cells of the stroma are also known to play an important role in creating favorable conditions for the tumor to spread [94,95]. However, it remains unclear, whether and to what extent non-epithelial cells influence the regulation of stemness and differentiation mechanism (Figure 4C), a topic which is currently an intensive focus of research. Defined niches of adult stem cells in the epithelial layer of the intestinal tract exhibit autonomous developmental and regulatory programs which are independent of the underlying parenchyma. Therefore, they can exert the complete repertoire of stem cell functions (long term propagation and differentiation) *in vitro* without the presence of non-epithelial cells [96]. It is possible, however, that CSCs evolved interaction strategies with their environment which provide them with an additional selective advantage and ensure unlimited growth.

## 13. Conclusions

The identification of fallopian tube epithelium as the tissue of origin of HGSC provides a basis for critical evaluation of the molecular mechanisms of pathology behind this deadly disease. In the present paper we have summarized both clinical and biological findings that point to the central role of the fallopian tube epithelial cells in the carcinogenesis of HGSC. Even though many important questions remain to be answered, including the exact mechanism of migration of the transformed fallopian cells to the ovary, embryological and genetic profiling shows clear evidence of the tight association between Fallopian tube and HGSC. Moreover, future studies are warranted to illuminate the initial cause of malignant transformation and the course of events which lead to the appearance of p53 mutation in tubal fimbriae, and hence the development of STICs. The regulatory mechanisms of epithelial renewal in the healthy tube should also be investigated, especially the existence of plasticity and pluripotent cells which could potentially lead to identification of the cancer initiating cells.

The elucidation of the pathways of carcinogenesis will have an immediate implication in the clinical management of BRCA mutation carriers, in terms of prophylactic salpingectomy with preservation of the hormonally active ovarian tissue. Of course, prior to implementation of this strategy as the standard of care in clinical practice, any remaining uncertainties about the origin of HGSC must be clarified. Multiple lines of evidence indicate that the epithelium of the fallopian tube constitutes an ideal model for studying the inflammatory and potentially infection-related basis of HGSC and also offer a platform for innovative screening assays of gene function profiling, as well as for testing the effects of new therapeutic compounds on cell transformation and growth. In particular, the biology of ovarian CSCs and mechanisms of chemoresistance acquisition are highly promising areas of research, which may provide new insights that could lead to a therapeutic and prognostic breakthrough of this fatal disease.
